# Supplementary material for: Natural Zeolites for the Sorption of Ammonium: Breakthrough Curve Evaluation and Modeling
Source: Molecules. 2023 Feb 7;28(4):1614. doi: 10.3390/molecules28041614 (PMC9965154; doi:10.3390/molecules28041614)
Supplement: Supplementary file 1 [file molecules-28-01614-s001.zip › molecules-2196274-supplementary-final.pdf]

molecules

Supplementary Materials

Natural Zeolites for the Sorption of Ammonium: Breakthrough Curve Evaluation and Modeling

Stephan Eberle, Viktor Schmalz, Hilmar Börnick and Stefan Stolte *

Institute of Water Chemistry, Technische Universität Dresden, 01062 Dresden, Germany
* Correspondence: stefan.stolte@tu-dresden.de; Tel.: +49-(0)351-463-32759

Supporting Information

S1 Isothermal Data

(a)

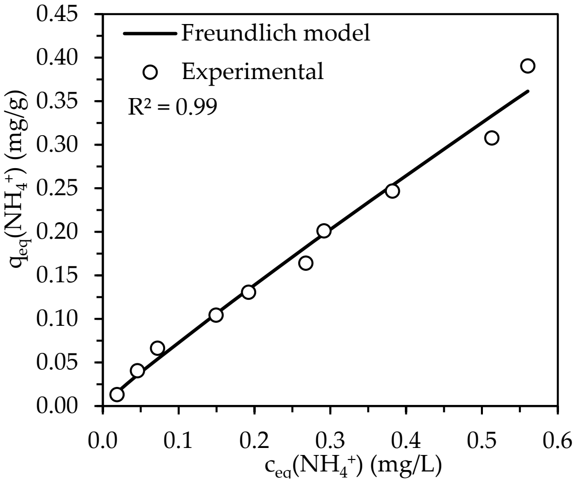

(b)

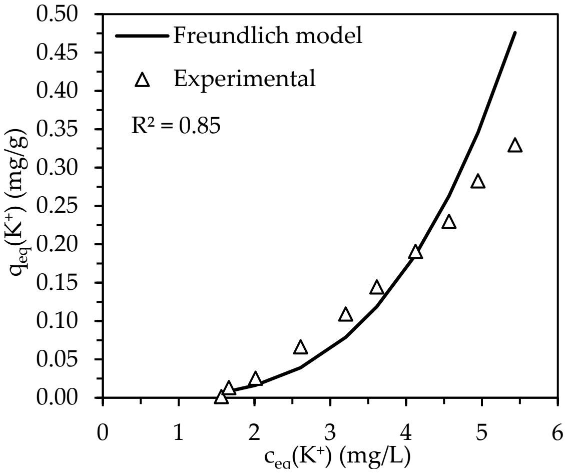

(c)

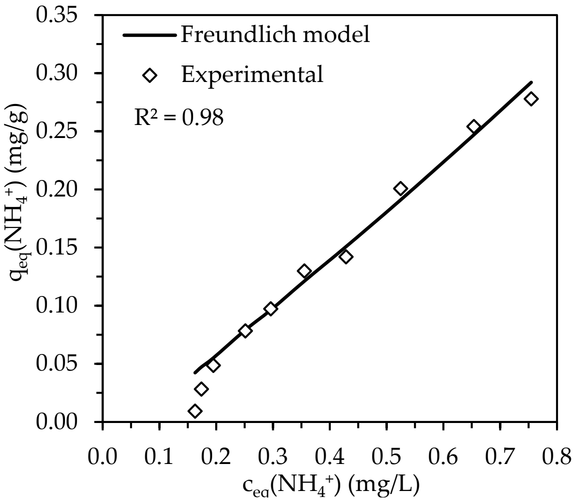

Figure S1. Experimental and Freundlich model isotherms (matrix: ultrapure water; t_{eq}: 21 d; pH_0: 5.5–6.2; (**a**): $c_0(NH_4^+)$ = 0.06–2.22 mmol/L; (**b**): $c_0(K^+)$ = 0.03–1.02 mmol/L; (**c**) $c_0(NH_4^+)$ = 0.06–2.22 mmol/L, $c_0(K^+)$ = 0.7 mmol/L; *w*/*v* = 100 g_Z/L, T = 22 °C; grain size: 0.5–0.8 mm; *n* = 2).

S2 Kinetic Data

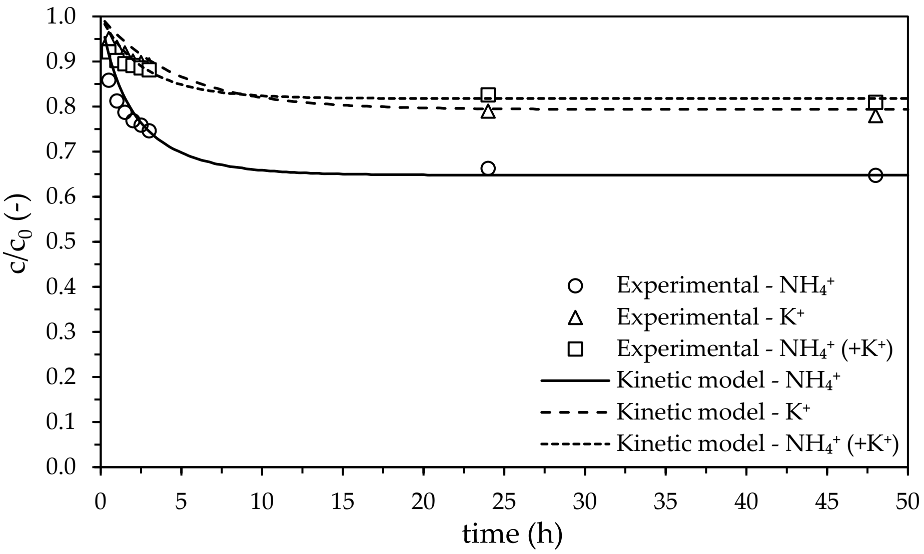

Figure S2. Experimental and modeled kinetic data (matrix: ultrapure water; t_{eq}: 48 h; pH_0: 5.5–6.2; ($c_0(NH_4^+)$ = 0.7 mmol/L, $c_0(K^+)$ = 0.7 mmol/L, $c_0(NH_4^+, K^+)$ = 0.7 mmol/L; *w*/*v* = 0.7 g_Z/L, T = 22 °C; grain size: 0.5–0.8 mm; *n* = 2).

S3 Thomas Model Evaluation in Ultrapure Water

Table S1. Determined Thomas model constants (matrix: ultrapure water).

**Water matrix**	**Thomas model constants**			
	**k_{Th} (L/h*mg)**	**a (mg/g)**	**b**	**R^2**
0.7 mmol/L NH_4^+	1.44 ± 9.73	2.24 ± 15	0.12 ± 0.83	0.98
0.7 mmol/L K^+	0.015 ± 0.62	92.76 ± 3723	6.42 ± 258	0.95
0.7 mmol/L NH_4^+/K^+	0.25 ± 1.76	13.04 ± 92	1.56 ± 11	0.99

S4 Influence of Natural Water Matrices

Table S2. General information to the investigated natural water matrices.

**Water matrices**	**EC [x] (µS/cm)**	**Sample description**	
Groundwater	560 ± 2.8	Date of sampling:	14.10.2022
		Sampling Location:	Sampling in 70 m distance to the Elbe river (50°57'56.6"N 13°55'25.5"E)
		Characteristics:	pH: 7.6; T = 14 °C
		Autoclaved:	25 min at 121 °C
Elbe water	462 ± 1.7	Date of sampling:	09.09.2022
		Sampling Location:	Elbe river in Dresden near the bank (51°04'19.0"N 13°43'21.1"E)
		Characteristics:	pH: 7.8; T = 17 °C
		Autoclaved:	25 min at 121 °C

[x] electrical conductivity

S5 Influence of Potassium and the DOC on the Thomas model constants

Table S3. Determined Thomas model constants (matrix: natural water).

**Water matrix**	**Thomas model constants**			
	**k_{Th} (L/h*mg)**	**a (mg/g)**	**b**	**R^2**
Tap water	0.04 ± 0.81	48.57 ± 946	3.22 ± 63.02	0.97
Elbe-/tap water (1:10)	0.15 ± 3.19	13.86 ± 293	1.03 ± 22.03	0.96
Groundwater	0.33 ± 10.48	6.14 ± 191	0.74 ± 23.19	0.94
Elbe river water	0.58 ± 16.62	3.35 ± 96	0.53 ± 15.41	0.96

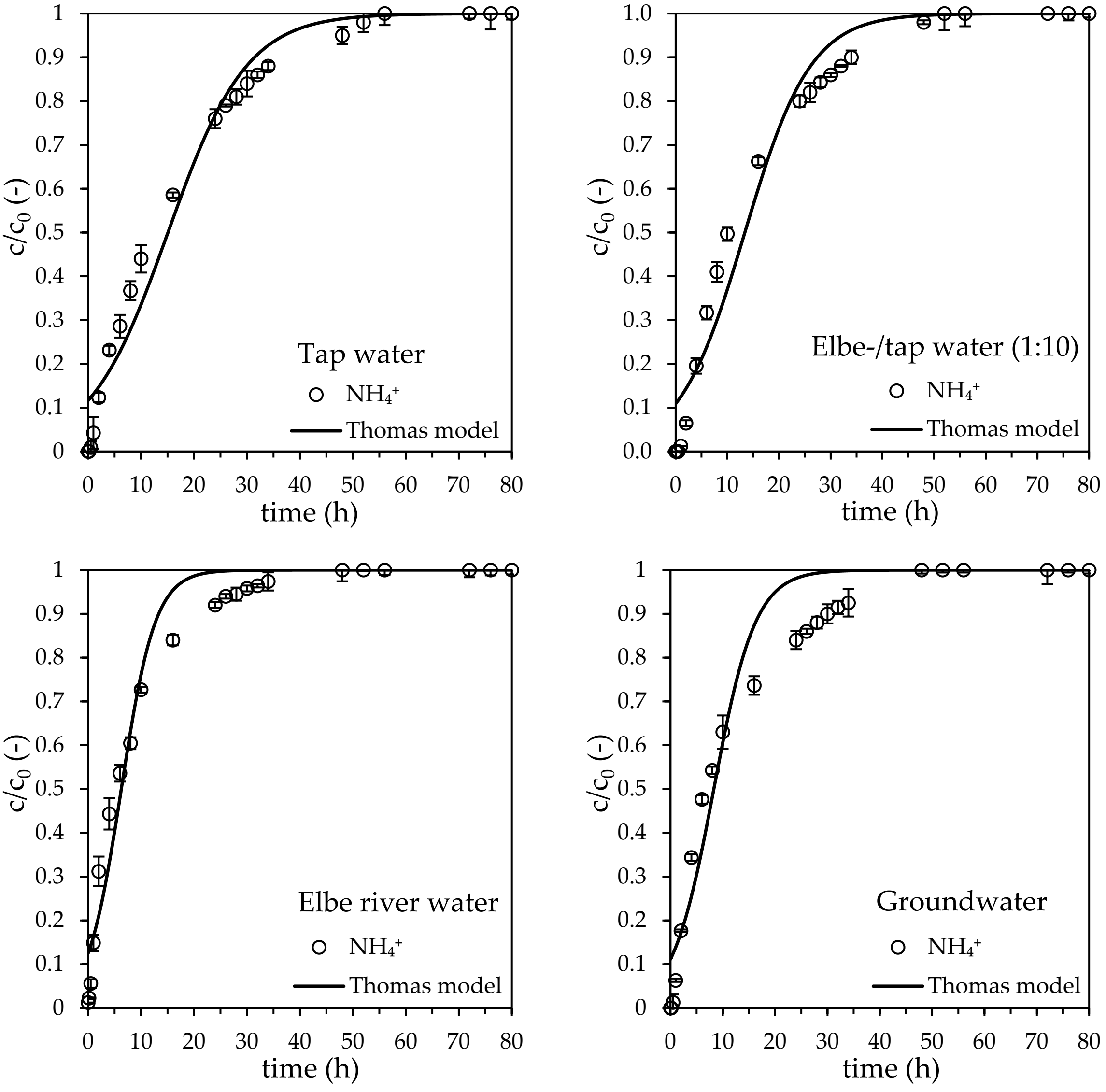

Figure S3: Experimental NH_4^+ breakthrough curves and modeling using the Thomas model.
